# Broadband Ge/SiGe quantum dot photodetector on pseudosubstrate

**DOI:** 10.1186/1556-276X-8-217

**Published:** 2013-05-08

**Authors:** Andrew Yakimov, Victor Kirienko, Vladislav Armbrister, Anatolii Dvurechenskii

**Affiliations:** 1 Rzhanov Institute of Semiconductor Physics, Siberian Branch of the Russian Academy of Sciences, Prospekt Lavrent’eva 13, Novosibirsk 630090, Russia

**Keywords:** Quantum dots, Silicon, Germanium, Interband transitions, Infrared photodetectors

## Abstract

We report the fabrication and characterization of a ten-period Ge quantum dot photodetector grown on SiGe pseudosubstrate. The detector exhibits tunable photoresponse in both 3- to 5- *μ*m and 8- to 12- *μ*m spectral regions with responsivity values up to about 1 mA/W at a bias of −3 V and operates under normal incidence radiation with background limited performance at 100 K. The relative response in the mid- and long-wave atmospheric windows could be controlled through the applied voltage.

## Background

There is an increasing need for sources and detectors for mid-infrared (IR) spectral region due to the broad range of medical and industrial applications such as measurement of skin temperature, detection of cancer or infection, air pollution monitoring, meteorological research, and remote temperature sensing. Quantum well infrared photodetectors (QWIPs) utilizing intersubband transitions have been successful in these applications [1]. The intersubband transition energy in the quantum well is easily tunable by varying the quantum well width and barrier height. Also, there is a potential for the fabrication of uniform detector arrays with large area. However, QWIPs have drawbacks such as intrinsic insensitivity to the normal incidence radiation and a relatively large dark current.

In the past several years, there has been a surge of interest in nanostructures that exhibit quantum confinement in three dimensions, which are known as quantum dots (QDs). With respect to quantum wells, the additional in-plane confinement of carriers and the peaked density of states in QDs lead to attractive properties in the mid-wave (3 to 5 *μ*m) and long-wave (8 to 12 *μ*m) IR regions where the Earth’s atmosphere has its major transmission windows [2]. The potential advantages of the quantum dot infrared photodetectors (QDIPs) as compared with two-dimensional systems are the following [3,4]: (1) increased sensitivity to normally incident radiation as a result of breaking of the polarization selection rules, so eliminating the need for reflectors, gratings, or optocouplers, (2) expected large photoelectric gain associated with a reduced capture probability of photoexcited carriers due to suppression of electron-phonon scattering, and (3) small thermal generation rate, resulting from zero-dimensional character of the electronic spectrum, that renders a much improved signal-to-noise ratio. Most of the demonstrations of QDIPs were achieved with III-V self-assembled heterostuctures. SiGe-based QDIPs represent another attractive type of the device due to its compatibility with the standard Si readout circuitry. At present, the most highly developed technology for fabricating arrays of SiGe-based QDs utilizes strain-driven epitaxy of Ge nanoclusters on Si(001) surface [5]. The photoresponse of Ge/Si heterostructures with QDs in the mid-wave atmospheric window was observed by several groups [6-10] and attributed to the transitions from the hole states bound in Ge QDs to continuum states of the Si matrix. Recently, we have reported on the photovoltaic operation of ten-period Ge/Si(001) QDIPs with Johnson noise-limited detectivity as high as 8×10^10^ cm Hz ^1/2^/W measured at photon wavelength (*λ*)=3.4 *μ*m and at 90 K under normal incidence IR radiation [11]. The cutoff wavelength at the low energy side of the responsivity of such QDIPs was limited to about 5 *μ*m.

There are only few works announcing the long-wave operation of detectors based on Ge/Si quantum dots [9,12-14]. Since the long-wavelength photoresponse in this system originates from the bound-to-bound intraband transitions, superior performance of such devices is unlikely, and one is obliged to seek another approach. Recently, the fabrication and characterization of a mid-IR QWIP on SiGe pseudosubstrate or virtual substrate (VS) were reported [15]. The use of the pseudosubstrate was found to lead to an increase in design freedom of quantum well devices and thus the possibility to improve their parameters. In this work, we demonstrate that the technologically important range between 8 and 12 *μ*m can be reached by the use of self-assembled Ge QDs grown on the relaxed Si _1−*x*_Ge _*x*_ layer (*x* = 0.4). The Ge/SiGe QDIP on SiGe VS displays a longer cutoff wavelength (approximately 12 *μ*m) and broader detection range as compared to conventional Ge/Si QDIPs due to smaller effective valence band offset at the Ge/Si _1−*x*_Ge _*x*_ interface.

## Methods

**Figure 1:**
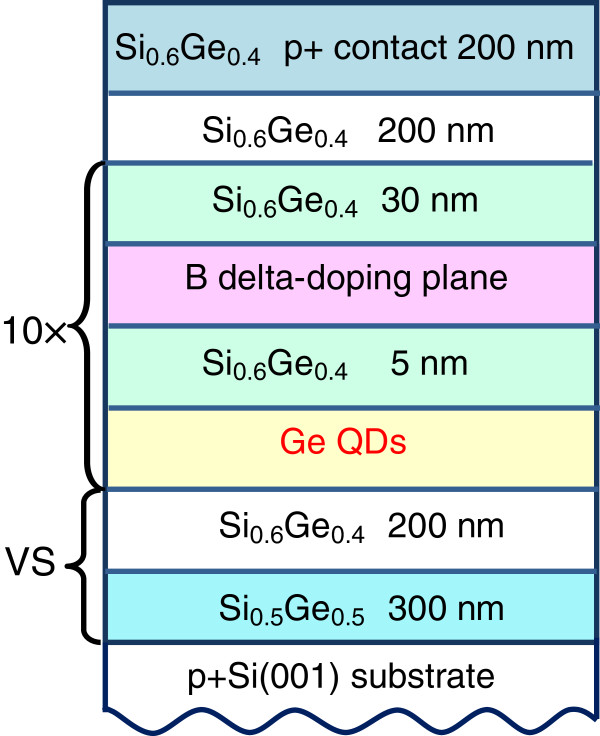
**Device structure of the QDIP on SiGe virtual substrate (VS).** The structure is that of a quantum dot infrared detector with ten layers of Ge QDs in a SiGe matrix.

Figure 1 shows schematically the structure of the detector discussed in this paper. The samples were grown by solid source molecular beam epitaxy on a (001)-oriented boron-doped p ^+^-Si substrate with resistivity of 0.004 *Ω* cm. For the fabrication of a virtual substrate with SiGe buffer layers, a method using a reverse grading by a two-step growth procedure was employed [16]. The fully relaxed Si _0.6_Ge _0.4_ VS was grown at 550°C on a Si _0.5_Ge _0.5_ layer which is only partially relaxed. The Si _0.5_Ge _0.5_ seed layer was deposited at low temperature of 350°C; its thickness *t* was such so as to keep a residual compressive strain and chosen to have a negligible lattice mismatch with the final Si _0.6_Ge _0.4_ VS. In our structure, *t* was adjusted to be 300 nm as determined from separate Raman measurements.

The active region of the device was composed of ten stacks of Ge quantum dots separated by 35-nm Si _0.6_Ge _0.4_ barriers grown on top of the virtual substrate. Each Ge QD layer consisted of a nominal Ge thickness of about 0.55 nm and formed by self-assembling in the Stranski-Krastanov growth mode at 500°C and at a growth rate of 0.02 nm/s. From scanning tunneling microscopy experiments with uncapped samples, we observed the Ge dots to be approximately 10 to 15 nm in lateral size and about 1.0 to 1.5 nm in height. The density of the dots is about 3 to 4 × 10^11^ cm ^−2^. The active region was sandwiched in between the 200-nm-thick intrinsic Si _0.6_Ge _0.4_ buffer and cap layers grown at 550°C. Finally, a 200-nm-thick p ^+^-Si _0.6_Ge _0.4_ top contact layer (3×10^18^ cm ^−3^) was deposited. The p-type remote doping of the dots was achieved with a boron *δ*-doping layer inserted 5 nm above each dot layer, providing after spatial transfer approximately three holes per dot. For vertical photocurrent (PC) measurements, the sample was processed into 700×700 *μ*m^2^ mesas by optical photolithography and contacted by Al/Si metallization. The bottom contact is defined as the ground when applying voltage to the detector.

The normal incidence photoresponse was obtained using a Bruker Vertex 70 Fourier transform infrared (FTIR) spectrometer (Ettlingen, Germany) with a spectral resolution of 5 cm ^−1^ along with a SR570 low-noise current preamplifier (Stanford Research Systems, Sunnyvale, CA, USA). The PC spectra were calibrated with a DLaTGS detector (SELEX Galileo Inc., Arlington, VA, USA). The dark current was measured as a function of bias *U*_*b*_ by a Keithley 6430 Sub-Femtoamp Remote SourceMeter (Cleveland, OH, USA). The devices were mounted in a cold finger inside a Specac cryostat (Orpington, Kent, UK) with ZnSe windows.

## Results and discussion

**Figure 2:**
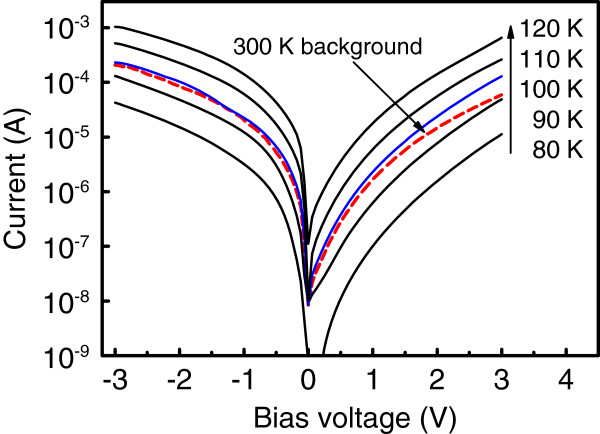
**The bias dependence of dark current measured at temperatures from 80 to 120 K.** The dashed line represents the response to a 300-K background radiation through the Dewar window (field of view = 53°). BLIP prevails at 100 K for negative bias voltage.

The detector dark current as a function of bias voltage, presented in Figure 2, was measured with a cold shield to eliminate background radiation for various temperatures from 90 to 120 K. Also shown in Figure 2 is the photocurrent measured at 80 K with the device illuminated from the 300-K background radiation (field of view = 53°). From these measurements, one can estimate the background limited performance (BLIP) temperature *T*_BLIP_ for our detector. Experimentally, *T*_BLIP_ can be estimated from comparing the dark current curves with the photocurrent characteristics obtained by allowing the 300-K radiation through the Dewar window [1]. In Figure 2, we can see that the current from the background radiation is equal to the dark current at 100 K and negative bias. This temperature is higher than that measured for Ge/Si QDIP [13] and GeSi/Si QWIP [17] operating in long-wave IR region and exceeds *T*_BLIP_ found for many n-type InAs QD-based detectors [18-21].

**Figure 3:**
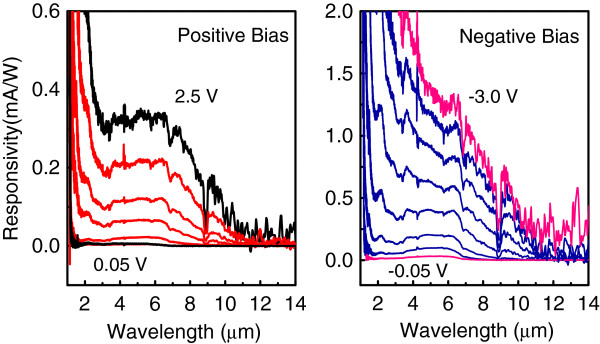
**Responsivity spectra under different applied biases of Ge/SiGe QDIP.** The applied voltages are ±0.05, ±0.1, ±0.5, ±1.0, ±1.5, ±2.0, ±2.5, and −3.0 V. The sample temperature is 90 K.

Figure 3 shows the normal incidence spectral response at 90 K for different bias voltages. At zero bias, no signal is observed implying the device operates in a photoconductive mode [22], and at biases just above 3.5 V, the signal becomes too noisy to detect PC. Ge/SiGe QDIP is of wide detection window with the cutoff wavelength of about 12 *μ*m instead of 5 to 6 *μ*m for Ge/Si QDIPs of similar device structure [11]. Since the sample in FTIR experiments is simultaneously exposed to a wide range of photon energies, the spectra may display additional transitions due to two-photon processes [9]. The near-infrared photons with energies larger than the SiGe bandgap create electrons and holes mostly in the SiGe barrier. The nonequilibrium holes diffuse from the SiGe bulk towards the Ge QDs and are accumulated in the dots. Then, by absorbing the mid-infrared photons, the photoexcited holes may contribute to the mid-infrared PC. To check this assumption, a 2.5- *μ*m optical low-pass filter was introduced in front of the sample to eliminate the photons which may cause band-to-band transitions in the Si and SiGe layers. The long-wave part of the photoresponse remains unchanged. Thus, we conclude that the observed redshift is a result of smaller effective valence band offset at the Ge/Si _1−*x*_Ge _*x*_ interface. By an analogy with the behavior of Ge/Si QDIPs [11], the near-infrared response at *λ*<2 *μ*m is ascribed to the interband transitions between the electrons in the *δ* valleys of SiGe layers and the holes at the *Γ* point of Ge QDs. The mid-infrared signal at *λ*>3 *μ*m is associated with the hole intraband transitions which involve the dot bound states.

**Figure 4:**
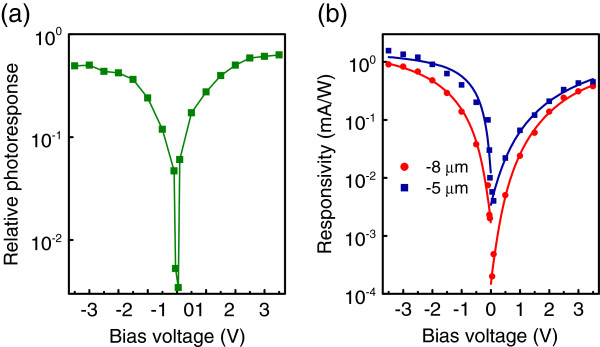
**Relative photoresponse and responsivity.** (**a**) Relative photoresponse of the device in long- and mid-wave regions. (**b**) Responsivity at *λ*=5 and 8 *μ*m as a function of applied bias. Solid curves are the best fit of experimental data to expression (1). The sample temperature is 90 K.

The bias voltage dependence of the relative photoresponse *R*_long_/*R*_mid_ is plotted in Figure 4a, where *R*_long_ is the PC integrated over the long-wave window from 8 to 12 *μ*m, and *R*_mid_ is the integral response in the mid-wave region from 3 to 5 *μ*m. As can be seen in Figure 4a, the relative intensity of the device response in major atmospheric windows can be strongly varied by changing the bias voltage. In mid-infrared region, at low bias, only the signal around 5 *μ*m is clearly visible, indicating excitation of holes into the valence band continuum states where the holes can easily reach the contact. As the applied voltage is increased, the PC at longer wavelength appears and grows rapidly, and at |*U*_*b*_|>2 V, both mid- and long-wave signals become comparable. We suppose that the long-wave photoresponse is caused by the excitation of holes to a shallow level confined in QD near the valence band edge with subsequent field-assisted tunneling through a barrier.

To check this interpretation, the voltage dependence of the mid-wave photoresponse (*λ* = 5 *μ*m) and long-wave PC (*λ* = 8 *μ*m) was analyzed separately. The inherent feature of tunneling mechanism of carrier escape is the exponential dependence of PC intensity *I* on the applied voltage. Finkman and co-workers [9] proposed a simple equation which follows from the WKB approximation:

(1) $$I=I_{0}\exp\left[-\frac{4}{3}\sqrt{\frac{2m^{*}}{\hbar^{2}}V_{B}^{3}}\frac{d}{q(U_{b}+U_{0})}\right],$$

where *I*_0_ is the intensity prefactor, *m*^∗^ is the hole effective mass, *V*_*B*_ is the tunneling barrier height, *d* is the contact separation, *U*_0_ is the built-in voltage, and *q* is the elementary charge. The results of the fitting analysis for both bias polarities are presented in Figure 4b by solid lines. It is clear that the 5- *μ*m PC is not characterized well by Equation 1. On the contrary, the theoretical curves show good agreement with the 8- *μ*m experimental data. From the best fit, we derive the barrier height *V*_*B*_=12 meV for negative bias and 19 meV for positive bias. The built-in voltage was found to be *U*_0_=0.68 and 0.94 V for *U*_*b*_<0 and *U*_*b*_>0, respectively. These values are typical for p-type Ge/Si QDIPs [9].

**Figure 5:**
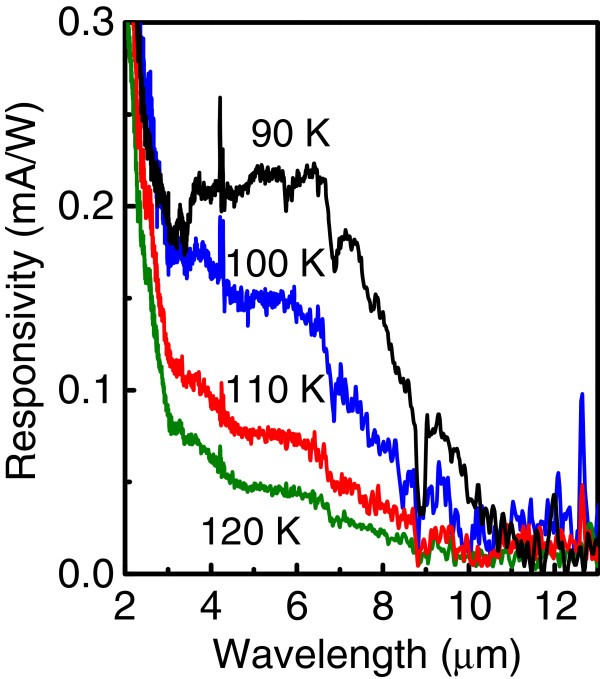
**Responsivity spectra measured at temperatures from 90 to 120 K.** The applied voltage is 2 V.

Figure 5 shows the spectral response measured with an applied voltage of 2 V in the temperature range of 90 to 120 K. The long-wave signal rapidly decreases at high temperatures because the probability of occupation of the dot excited states increases with temperature thus blocking the interlevel transitions.

## Conclusions

In summary, we report a normal incidence broadband mid-IR Ge/SiGe quantum dot photodetector on SiGe virtual substrate with a background limited performance at 100 K. The detector exhibits photoresponse in both the 3- to 5- *μ*m and 8- to 12- *μ*m spectral regions. The operating wavelength range of the device can be varied via the bias voltage. The long-wave responsivity measured at 90 K (approximately 1 mA/W) is higher or comparable to previously reported values for Ge/Si QDIPs [13,14] and SiGe/Si QWIPs [23] at much lower temperatures (10 to 20 K). The proposed device is compatible with the existing Si readout circuitry and suitable for monolithic focal plane array applications.

## Abbreviations

BLIP; background-limited-performance; FTIR: Fourier transform infrared; IR: infrared; PC: photocurrent; QD: quantum dot; QDIP: quantum dot infrared photodetector; QWIP: quantum well infrared photodetector; λ: photon wavelength.

## Competing interests

The authors declare that they have no competing interests.

## Authors’ contributions

AY conceived and designed the experiment, carried out the photocurrent measurements, coordinated the study, and drafted the manuscript. VK and VA prepared the samples using molecular beam epitaxy and photolithography techniques. AD supervised the project work. All authors read and approved the final manuscript.
